# Left-Right Asymmetry Is Required for the Habenulae to Respond to Both Visual and Olfactory Stimuli

**DOI:** 10.1016/j.cub.2014.01.016

**Published:** 2014-02-17

**Authors:** Elena Dreosti, Nuria Vendrell Llopis, Matthias Carl, Emre Yaksi, Stephen W. Wilson

**Affiliations:** 1 Department of Cell and Developmental Biology, University College London, Gower Street, London WC1E 6BT, UK; 2 NERF, Kapeldreef 75, 3001 Leuven, Belgium; 3 KU Leuven, Kapeldreef 75, 3001 Leuven, Belgium; 4 Department of Cell and Molecular Biology, Medical Faculty Mannheim, University of Heidelberg, Ludolf-Krehl-Strasse 13–17, 68167 Mannheim, Germany; 5 VIB, Kapeldreef 75, 3001 Leuven, Belgium

## Abstract

Left-right asymmetries are most likely a universal feature of bilaterian nervous systems and may serve to increase neural capacity by specializing equivalent structures on left and right sides for distinct roles [1]. However, little is known about how asymmetries are encoded within vertebrate neural circuits and how lateralization influences processing of information in the brain. Consequently, it remains unclear the extent to which lateralization of the nervous system is important for normal cognitive and other brain functions and whether defects in lateralization contribute to neurological deficits [2]. Here we show that sensory responses to light and odor are lateralized in larval zebrafish habenulae and that loss of brain asymmetry leads to concomitant loss of responsiveness to either visual or olfactory stimuli. We find that in wild-type zebrafish, most habenular neurons responding to light are present on the left, whereas neurons responding to odor are more frequent on the right. Manipulations that reverse the direction of brain asymmetry reverse the functional properties of habenular neurons, whereas manipulations that generate either double-left- or double-right-sided brains lead to loss of habenular responsiveness to either odor or light, respectively. Our results indicate that loss of brain lateralization has significant consequences upon sensory processing and circuit function.

## Highlights

• Habenular neuron responses to light and odor stimuli are lateralized
• Lateralized habenular light responses depend upon the eyes
• Loss of brain asymmetry leads to a loss of either light or odor responses

## Graphical Abstract

**Figure:**
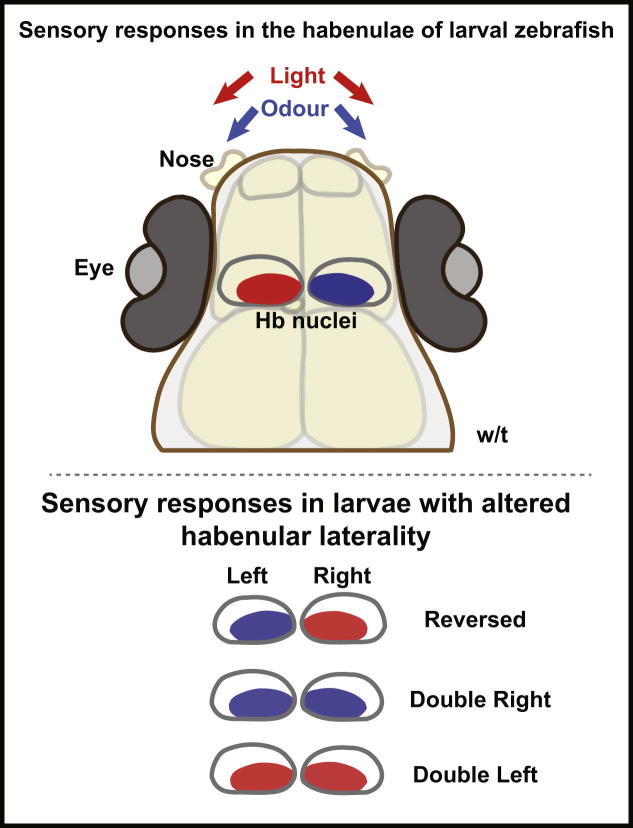

## Teaser

Dreosti et al. show that sensory responses to light and odor are lateralized in the larval zebrafish habenula and that loss of brain asymmetry leads to a loss of responses to visual or olfactory stimuli, suggesting that brain lateralization may play a significant role in sensory processing and circuit function.

## Results and Discussion

### Responses to Visual and Odor Stimuli Are Lateralized in the dHb and Segregated in the IPN

**Figure 1:**
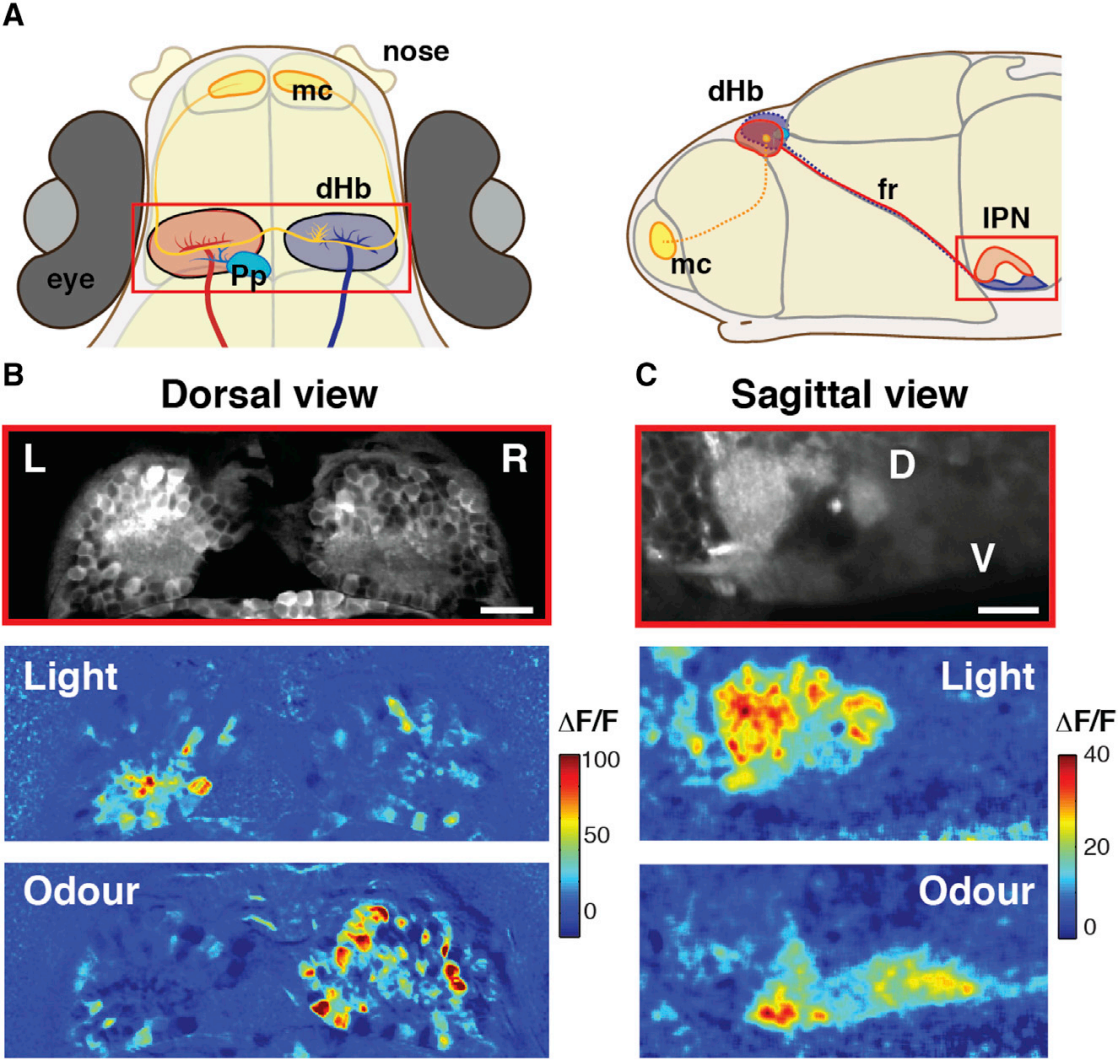
Responses to Visual and Odor Stimuli Are Lateralized in the dHb and Segregated Dorsoventrally in the IPN (A) Schematic dorsal and sagittal views of 4 days postfertilization (dpf) zebrafish showing left (red) and right (blue) dHb nuclei and their asymmetric afferents from olfactory mitral cells (yellow) arborizing in the right dHb nucleus and parapineal neurons (cyan) arborizing in the left dHb nucleus. Neurons of the left dHb predominantly innervate the dorsal IPN, while neurons of the right dHb innervate the ventral IPN. (B) Example of a two-photon image of a single z plane of the dHb (14 μm below the skin) of a Tg(*elavl3*:GCaMP5G) 4 dpf fish (top) and corresponding color-coded calcium signals that are LR lateralized in dHb neurons in response to a nonlateralized presentation of light (middle) and odor (bottom) stimuli. Each panel is an average of two stimulus trials. The relative change in fluorescence (ΔF/F) is expressed as a percentage. (C) Example of a two-photon image of a lateral view of the IPN of a Tg(*elavl3*:GCaMP5G) 4 dpf fish (top) and corresponding color-coded calcium responses to light (middle) and odor (bottom). Each panel is an average of three trails. Responses to light are predominantly localized in the dorsal IPN, while those to odor are localized in the ventral IPN. Note that the dorsal IPN has a higher basal fluorescence (as does the left dHb nucleus). Scale bars, 20 μm. D, dorsal; V, ventral; fr, fasciculus retroflexus; IPN, interpeduncular nucleus; dHb, dorsal habenulae; L, left; R, right; mc, mitral cells; Pp, parapineal. See also Figure S1.

Cognitive and behavioral left-right (LR) asymmetries are common, but the neurons that underlie these asymmetries are, in most cases, poorly understood in terms of their development, circuitry, and function [1]. The epithalamus shows evolutionarily conserved asymmetries within both the pineal complex and the adjacent habenular nuclei [3] and is currently the most-tractable structure in which to study the development of neuroanatomical asymmetries in vertebrates [1]. Asymmetric patterns of gene expression and efferent projections of the left and right dorsal habenulae (dHb) [4–8], coupled with their asymmetric afferent input [9–11], suggest that left and right dHb neurons may also show functional asymmetries (Figure 1A and Figure S1 available online). To determine whether this is the case, we presented visual and olfactory sensory stimuli to restrained zebrafish larvae and quantified whether there were asymmetries in GCaMP5G [12] responses between dHb neurons on the left and right. Light stimuli were presented with a red light-emitting diode (LED; 625 nm) coupled to an optic fiber positioned in front of the fish. The odor stimulation was a solution containing 1mg/ml dried food dissolved in water, filtered, and delivered in front of the fish via a high-performance liquid chromatography (HPLC) valve.

**Figure 2:**
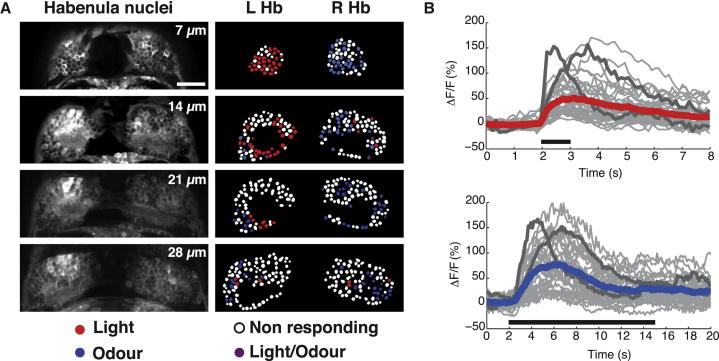
Functional Responses of Habenular Neurons to Visual and Odor Stimuli (A) Raw images of four z planes (at 7, 14, 21, and 28 μm depth from surface) of the dHb of a 4 dpf Tg(*elavl3*:GCaMP5G) wild-type fry. In the right panels, dHb neuron cell bodies, indicated as regions of interest (ROIs), are color coded in red, blue, violet, or white dependent upon their response to light, odor, both, or neither, respectively. To quantify the number of dHb neurons responding to light and odor, we analyzed the two most-dorsal planes (at 7 and 14 μm) that contained the majority of responding neurons. Only neurons with amplitude responses greater than 2 SDs from the baseline were considered as responding neurons. Neurons that showed inhibition of activity in response to stimulus presentation are not shown. Scale bar, 20 μm. (B) Graphs showing the time courses (averages of two stimulus presentations) of all individual neurons within one imaging plane, at 14 μm depth, to light (top) and odor (bottom) stimuli. Individual responses are shown in gray, the average of all responses is colored red or blue, and two neurons demonstrating the distinct kinetics of the sensory responses are highlighted in dark gray. The durations of the light and odor stimulation are indicated by the black bars; note the difference in time scale. See also Figure S2.

Two-photon calcium imaging of neuronal activity of dorsal habenular neurons (a representative example of a 4-day-old fish is shown in Figure 1B) showed that the majority of cells with sensory responses are functionally lateralized. Most neurons that respond to a binocular light stimulus are located on the left, and most neurons that respond to an odor stimulus are located on the right, with very few neurons responding to both modalities (Figure 2A). Imaging through the full depth of the Hb in individual fish showed that lateralized sensory responses were predominantly observed in the most-dorsal domain of the habenulae, corresponding to the dHb, (Figure 2A). The ventral habenulae showed fewer sensory responses with more symmetric distribution. The time courses of the responses of neurons in a single plane of the dHb (at 14 μm depth) to light or odor demonstrate their broad range of evoked response kinetics (Figure 2B). Lateralization of odor responses is maintained into adulthood [13], although the complexity of responses increases significantly [14].

The dHb is constituted by lateral (dHbl) and medial (dHbm) subnuclei containing neurons that send efferent projections to the dorsal (dIPN) and ventral (vIPN) interpeduncular nuclei, respectively [4, 6, 8]. To assess whether the dHb neurons that respond to the light and odor stimuli belong to dHbl or dHbm subclasses, we measured IPN responses to light and odor.

**Figure 3:**
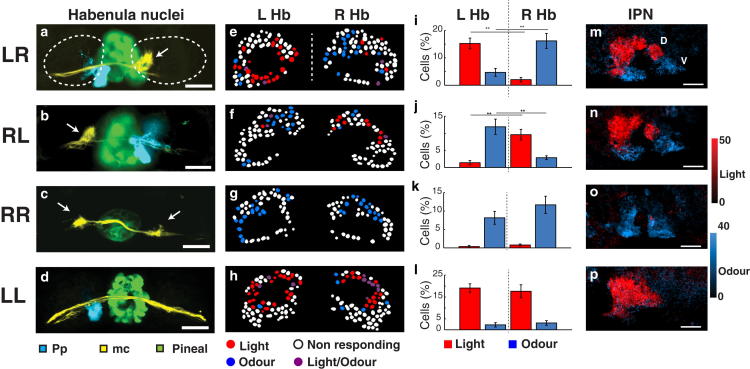
Developmental Disruption of dHb Asymmetry Leads to Loss of Sensory Responses to Light or Odor (A–D) Confocal images of the epithalamus of wild-type (A), cold-shocked (B), parapineal-ablated (C), and IWR1-treated (D) Tg(*foxD3*:GFP × *lhx2a*:Gap43-YFP) 4 dpf fish showing normally lateralized (LR), reversed (RL), double-right (RR), and double-left (LL) epithalami. Olfactory mitral cell projections (white arrows) terminate in the right dHb nucleus in the wild-type brain (A), terminate in the left dHb in the reversed brain (B), are bilateral in the double-right brain (C), and are reduced/absent in the double-left brain (D). Parapineal neuron projections (pseudocolored cyan) terminate in the left dHb in the wild-type and double-left brain (A and D), terminate in the right dHb in the reversed brain (B), and are reduced/absent in the double-right brain (C). (E–H) Examples of single z planes of the dHb (as in Figures 1B and 2A) of wild-type (E), cold-shocked (F), parapineal-ablated (G), and IWR1-treated (H) Tg(*foxD3*:GFP × *lhx2a*:Gap43-YFP) 4 dpf fish as in (A)–(D) showing lateralization of neuronal responses to light and odor. Neuronal cell bodies are shown as ROIs color coded in red, blue, violet, or white depending on whether they responded to light, responded to odor, responded to both light and odor, or were nonresponding. Only neurons with amplitude responses greater than 2 SDs from the prestimulus baseline are considered as responding. Neurons that showed an inhibitory response are not shown. (I–L) Bar charts showing the percentages of light (red)- versus odor (blue)-responding neurons (normalized to the total number of dHb neurons) in the left and right dHb nuclei for two sampled planes (at depths of 7 and 14 μm) in multiple fish of each of the classes shown in (A)–(D). For the wild-type (I), an average of about 17% of neurons respond to light 20% to odor and 1% to both (n = 8 fish). The LR difference in light responses was significant (p = 4 × 10^−5^), as was the LR difference in odor responses (p = 5 × 10^−4^; ^∗∗^p < 0.001, Student’s t test). For reversed fish (J), about 10% of dHb neurons respond on average to light, 14% to odor, and 0.6% to both. LR difference responses was significant to light (p = 0.0056) and odor (p = 0.0072) (n = 7 fish). In double-right-brained fish (K), 21% of neurons were odor responding, with light-responding neurons reduced to 0.5% and those responding to both to 0.2% (n = 8 fish), whereas double-left-brained fish (L) showed an increased percentage of neurons responding to light (35%), a reduced number responding to odor (3%), and 3% responding to both (n = 6 fish). Error bars indicate the SEM. (M–P) Sagittal views of the IPN in Tg(*elavl3*:GCaMP5G) 4 dpf fish showing excitation in response to light (red) and odor (blue), color coded depending on their ΔF/F responses. In the wild-type (M) and reversed (N) fish, light and odor responses are localized in the dorsal and ventral IPN, respectively. In the IPN of the double-left-brained fish (P), virtually all IPN responses were upon light stimulation, whereas in the double-right-brained fish (O), virtually all IPN responses were upon odor stimulation. Scale bars, 20 μm. D, dorsal; V, ventral; Pp, parapineal; mc, mitral cell axons and terminals; LHb, left habenula nucleus; R Hb, right habenula nucleus. See also Figures S3 and S4.

Calcium imaging of the IPN showed that light responses were predominantly localized to the dIPN, whereas odor responses were localized to the vIPN (Figures 1C, 3M, and S3). Therefore, we conclude that visual information processed by a subset of dHbl neurons predominantly in the left nucleus is transmitted to the dIPN, whereas odor information from a subset of dHbm neurons predominantly in the right nucleus is sent to the vIPN. The habenulae and IPN are core components of an evolutionarily conserved output pathway in the brain [15], and previous studies have shown that neurons in the dIPN and vIPN project to distinct targets [16], potentially differentially modulating behavioral responses [17]. Consequently, our results indicate that sensory information of different modalities is processed asymmetrically in the zebrafish habenulae and feeds into distinct output pathways between left and right sides of the brain.

### Disruption of dHb Asymmetry Leads to Loss of Appropriate Sensory Responses in the Habenulae

It is largely unclear whether and how structural asymmetries are related to functional asymmetries in vertebrates. For instance, humans with situs inversus totalis show reversal of some structural asymmetries in the brain but appear to usually show left-hemisphere dominance for language and right handedness [18, 19]. To explore the relationship between the lateralized neuroanatomy and function of dHb neurons, we imaged activity of dHb neurons in cold-shocked fish that show reversed laterality in the brain. Cold shock disrupts lateralization of Nodal signaling, which can result in reversal of both lateralized gene expression in dHb neurons [20] and lateralized afferent innervation of the dHb by mitral cells and parapineal neurons (Figures 3A and 3B).

In contrast to the wild-type (Figures 3E and 3I), embryos with reversed epithalamic laterality exhibit light responses predominantly localized to the right dHb and odor responses to the left dHb, correlating with the LR reversal of dHb innervation by parapineal and mitral cell axons (Figures 3F, 3J, and S2). Consequently, LR reversal of the early genetic pathways mediating brain lateralization results in a LR reversal in the response of subsets of dHb neurons to light and odor. Although dHb responses to light and odor were reversed, IPN responses were unaffected, with light responses still being localized to the dIPN (albeit now due to activation of right rather than left-sided dHb neurons) and odor responses being localized to the vIPN (Figures 3N and S3). This is consistent with the LR-reversed but dorsoventrally appropriate innervation of the IPN by dHb axons in fish with reversed brain laterality [4, 8].

The possibility that neuroanatomical asymmetries enhance cognitive processes by specializing circuits on the left and right [1] is consistent with our observation of lateralization of sensory responses to light and odor in the dHb. To determine whether sensory processing may be disrupted when brain asymmetry is lost, we assessed neuronal activity in fish that either showed a “double-right” phenotype, in which most dHb neurons differentiate with the dHbm character typical of the majority of right-sided neurons, or a “double-left” phenotype, in which most neurons have the dHbl phenotype typical of most left-sided neurons. The parapineal is required for elaboration of left-sided character of dHb neurons, and parapineal-ablated fish show largely double-right-sided habenulae [5, 6, 11], in which both left and right nuclei are innervated by mitral cells [21] (Figure 3C). Embryos with enhanced Wnt signaling also show an overtly double-right phenotype [22]. Furthermore, we were able to generate a habenular “double-left” phenotype, in which many neurons on the right expressed markers typical of neurons on the left, by transiently inhibiting Wnt signaling pharmacologically with IWR-1 [23]. This compound suppresses Wnt signaling and causes most dHb neurons to express markers of lateral subnuclear identity typical of left-sided dHb neurons when applied around the time of birth of the neurons (M.C. and S.W.W., unpublished data; Figure S4). The habenulae of these double-left-brained fish showed a strong reduction of mitral cell innervation and bilaterally symmetric patterns of expression of markers normally lateralized to the left dHb nucleus (Figure 3D; M.C. and S.W.W., unpublished data; Figure S4).

Loss of asymmetry in the epithalamus resulted in the loss of either odor or light responses within the larval dHb and IPN depending on whether the habenulae showed double-left or double-right character. In double-right brains, odor responses were bilateral in the dHb and robust in the vIPN, whereas light responses were severely reduced in both the dHb and dIPN (Figures 3G, 3K, 3O, S2, and S3). Conversely, in double-left brains, very few dHb neurons responded to odor, whereas neurons on both left and right responded to light (Figures 3H, 3L, and S2). Consequently, light-dependent responses in the dIPN were robust, whereas olfactory responses in the vIPN were almost absent (Figures 3P and S3). These results indicate that in contrast to reversal, loss of asymmetry potentially has much more severe consequences on the processing of lateralized sensory information, given that dHb responses to specific sensory modalities can be lost.

### Retinal Input Contributes to the Visual Responses of dHb Neurons

Lateralization of olfactory responses is well documented in invertebrates [24], and in zebrafish, the innervation of the right dHb by olfactory bulb mitral cells [10] most likely underlies the mechanism by which olfaction is lateralized in the epithalamus. Indeed, mitral cell innervation of the dHb is bilateral in double-right brains and reduced or absent in double-left brains (Figures 3C and 3D). Zebrafish, as probably do all other vertebrates, show preferential eye use for different tasks [25], but it is unclear how such visual asymmetries are encoded within the brain and whether there is any involvement of the habenulae. The parapineal is photoreceptive, and its innervation of the left dHb may contribute to lateralization of light responses. However, in double-left brains, parapineal innervation remains unilateral (Figure 3D), yet right-sided dHb neurons respond to light, suggesting that the eyes may be important for this response. To assess the source of visual input to the asymmetric functional responses of the dHb, we ablated the eyes, either genetically or physically.

**Figure 4:**
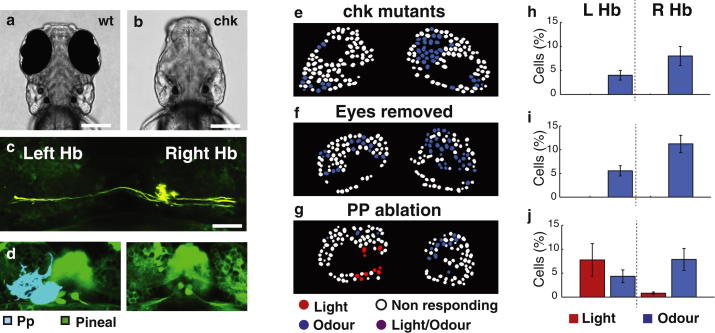
Asymmetric Responses of left dHb Neurons to Light Are Dependent upon the Eyes (A and B) Dorsal views of live wild-type (A) and *chk*^ne2611^ mutant (B) fish at 4 dpf. (C) Dorsal view of the dHb nuclei in a *chk*^ne2611^ mutant showing that mitral cells inputs remain asymmetrically directed to the right dHb. (D) Dorsal views of the epithalamus of a Tg(*elavl3*:GCaMP5G) 3 dpf fish prior (left) and 4 dpf fish subsequent (right) to ablation of the parapineal (pseudocolored blue). (E–G) Images of color-coded dHb neuron responses to light (red) and odor (blue) in single z plane of a 4 dpf Tg(*foxD3*:GFP × lhx2a:Gap43-YFP) *chk*^ne2611^ mutant fish (E), a fish in which eyes were surgically removed at 1 dpf (F), and a Tg(*foxD3*:GFP × *elavl3*:GCaMP5G) embryo with late ablation of the parapineal (G). (H–J) Bar graphs showing average dHb neuron responses to light and odor for *chk* mutant (H) eye-ablated (I) and parapineal-ablated (J) fish. Fish without eyes show loss of dHb visual responses, whereas fish without a parapineal still show visual responses. For *chk*^ne2611^ mutant and eye-ablated fish, an average of 12% (n = 6 fish) and 16% (n = 6 fish), respectively, of neurons responded to odor. For late parapineal-ablated fish (n = 3 fish), dHb neurons show an asymmetric distribution of responses to light (9% of neurons) and odor (11% of neurons) comparable with that of the wild-type. Error bars indicate the SEM. Scale bars, 20 μm. Hb, habenulae; Pp, parapineal; LHb, left habenula nucleus; R Hb, right habenula nucleus.

Experiments in which either retinal or parapineal input was removed showed that visual input from the eyes is more important for lateralized responses in the dHb than from the parapineal. Both wild-type embryos in which eyes were ablated and *chk*^ne2611^ mutants in which eyes do not form [26] (Figure 4A–4C) failed to show robust visual responses in dHb neurons (Figures 4E, 4F, 4H, and 4I). In contrast, embryos in which the parapineal was ablated subsequent to its role in elaboration of dHbl neuronal character (Figure 4D) still showed light responses in the left dHb (Figures 4G and 4J). Even when light responses were absent, we still observed robust and lateralized responses to odor stimuli (Figures 4H and 4I). These experiments raised the possibility that visual sensory input may not be necessary for the establishment of functional asymmetries in the dHb (Figures 4E–4J). To further investigate the role of sensory experience, we analyzed the visual and olfactory responses of wild-type fry and fry reared in a dark environment (Figure S5). No overt differences in functional asymmetries were observed, consistent with previous studies showing that light is not needed for establishment of asymmetric gene expression [27]. To test the contribution of olfactory input to the establishment of functional asymmetries, we compared visual responses in fish for which the olfactory pits were ablated at 1 dpf, prior to habenular innervation by mitral cells, and, again, no overt change in the asymmetry of visual responses was observed (Figure S6). These results suggest that the initial functional differentiation of the dHb is achieved independently of sensory input and largely through the same genetic patterning mechanisms believed to underlie the establishment and maintenance of anatomical asymmetries.

Our study has revealed a striking asymmetry in response of habenular neurons to sensory stimuli and has shown that perturbations disrupting the specification of brain asymmetry result in the loss of ability of this region of the brain to respond appropriately to visual or olfactory cues. Furthermore, we found that peripheral inputs from the retina and olfactory pits are not necessary to establish functionally asymmetric circuitry, whereas the light-sensitive parapineal, although not the most-important source of visual input, is required for the establishment of lateralized sensory responses in the dHb. Various neurological conditions are associated with abnormalities in the lateralization of brain activity [2, 28, 29], but it is unclear whether these abnormalities are a cause or consequence of disease. This study raises the possibility that defects in the establishment of brain lateralization could indeed be causative of cognitive or other symptoms of brain dysfunction.

## Experimental Procedures

### Fish Lines and Maintenance

Maintenance and use of animals conformed with local ethical and licensing rules. Zebrafish (Danio rerio) larvae obtained by natural spawning from the wild-type, *mitfa*^w2/w2^ [30], Tg(*elavl3*:GCaMP5G)^2^ [12], Tg(*foxD3*:GFP)^zf104^ [11, 31], Tg(*flh*:EGFP)^U711^ [11], Tg(*lhx2a*:Gap43YFP)^zf177^ [10], and *chk* ^ne2611^ [26] were reared and staged according to standard procedures [32]. For wild-type fish, 0.002% phenylthiourea (PTU) was added to the fish water from 24 hours postfertilization (hpf) to inhibit pigment formation.

### Temperature, Drug Treatments, and Surgical Lesions

For obtainment of reversal of epithalamic laterality in Tg(*foxD3*:GFP); Tg(*elavl3*:GCaMP5G) larvae, embryos were shifted from 28°C to 22°C at the tailbud stage for 3–4 hr [20]. Efficacy of randomization of laterality was determined by assessment of parapineal position at 3–4 dpf. For obtainment of embryos with epithalami showing double-left laterality, dechorionated Tg(*elavl3*:GCaMP5G) 32 hpf embryos were treated with 0.1 μM IWR1-endo [23] dissolved in E3 medium containing 1% DMSO for 12 hr. The Wnt signaling inhibitor IWR-1 stabilizes Axin, which is an essential component of the β-catenin degradation complex, and reduces the amount of β-catenin available for Wnt signaling. The compound suppresses Wnt signaling and causes most dHb neurons to adopt a lateral subnuclear identity typical of left-sided dHb neurons. Consequently, it induces a double-left phenotype in the epithalamus (U. Hüsken, S.W.W. and M.C., unpublished data). Eye removal was performed with a sharpened tungsten needle in 1 dpf embryos as described in Zhang and Leung [33]. Parapineal ablation was performed as in Concha et al. [11] and Gamse et al. [5], with the exception that a two-photon laser was used for cell ablation (see below) and that some ablations were performed at 3 dpf after migration of the parapineal to the left side of the brain.

### Functional Imaging and Sensory Stimulation

Animals were anaesthetized with 0.02% MS222, injected with α-Bungarotoxin (1 mg/ml), mounted in 1.5% low-melting agarose in a 35mm diameter imaging dish, and imaged with a laser scanning 7 MP upright microscope (Zeiss). For odor stimulation, the agarose around the mouth and nose was removed with a scalpel to provide access to the nose. The odor stimulus was prepared by addition of 50 mg of standard dried fish food (size 000, ZM Fish Food) in 50 ml of artificial fish water (1.2 g of Instant Ocean sea salt per 20 liters of water), dissolving for 1 hr, and filtering of the solution with a 22 μm filter. Odor stimuli were delivered for 15 s via a tube positioned in front of the fish and controlled by an HPLC valve (Rheodyne). Light stimulation was delivered via an optical fiber (200 μm outer diameter, BFL22-200, ThorLabs) directly coupled to a red LED (LZ1-00R105, LedEngin; 625 nm wavelength). The end of the fiber was immersed in the chamber and positioned approximately 1 cm directly in front of the fish. The light stimulus was a square-wave flash of 1 s duration with an intensity of 450 μW. Functional responses of Tg(*elavl3*:GCaMP5G) or Tg(*foxD3*:GFP); Tg(*elavl3*:GCaMP5G) larvae to sensory stimulation were imaged with a two-photon laser scanning system (LSM 7 MP upright with 20× water immersion objective, LD Plan-Apochromat: 1.0 numerical aperture, Zeiss) at 8.26 Hz in the Hb and 4.13 Hz in the IPN. See the Supplemental Experimental Procedures for data analysis techniques.

### Laser Ablation and Confocal Imaging

Two-photon laser ablation of parapineal precursors at 28–32 hpf or the parapineal nucleus at 3 dpf in Tg(*foxD3*:GFP); Tg(*flh*:eGFP); Tg(*elavl3*:GCaMP5G) transgenic embryos was performed using a laser scanning system (LSM 7 MP upright Zeiss) equipped with a 20× water immersion objective (Zeiss LD Plan-Apochromat: 1.0 numerical aperture; 920 nm wavelength). Only larvae showing complete absence of parapineal cells at 4 dpf were used for experiments. For clarity in the figures, the parapineal nucleus was manually pseudocolored in cyan using ImageJ.

### Data analysis

All data analysis was performed using custom software written in MATLAB (Mathworks). For analysis of responses from single Hb neurons, all frames acquired from a specific depth plane were first registered. Somata from individual neurons were segmented into distinct ROIs using a previously described algorithm [34]. Fluorescence time courses for each neuron were averaged across three stimulus trials, and baseline fluorescence before stimulus onset was subtracted. Cells were classified as “responding” when the change in fluorescence within a time window after stimulation (window size: 0.6 s for light, 4 s for odor) exceeded 2 SDs above the SD measured during the baseline period.

For analysis of the spatial distribution of sensory responses in the IPN, aligned movies of the same sagittal plane were prepared. The DF/F time course was computed for each pixel, and the pixel was classified as responsive if it exceeded 10% DF/F during stimulation (same time windows as for Hb responses) with respect to baseline. The light- and odor-responding pixels within a user-defined rectangular region surrounding the whole IPN were binned into 20 equally sized horizontal rectangles spanning the ventral-dorsal axis of the IPN.

## References

[1] Concha M.L., Bianco I.H., Wilson S.W. (2012). Encoding asymmetry within neural circuits. Nat. Rev. Neurosci..

[2] Toga A.W., Thompson P.M. (2003). Mapping brain asymmetry. Nat. Rev. Neurosci..

[3] Concha M.L., Wilson S.W. (2001). Asymmetry in the epithalamus of vertebrates. J. Anat..

[4] Aizawa H., Bianco I.H., Hamaoka T., Miyashita T., Uemura O., Concha M.L., Russell C., Wilson S.W., Okamoto H. (2005). Laterotopic representation of left-right information onto the dorso-ventral axis of a zebrafish midbrain target nucleus. Curr. Biol..

[5] Gamse J.T., Thisse C., Thisse B., Halpern M.E. (2003). The parapineal mediates left-right asymmetry in the zebrafish diencephalon. Development.

[6] Bianco I.H., Carl M., Russell C., Clarke J.D.W., Wilson S.W. (2008). Brain asymmetry is encoded at the level of axon terminal morphology. Neural Dev..

[7] Concha M.L., Burdine R.D., Russell C., Schier A.F., Wilson S.W. (2000). A nodal signaling pathway regulates the laterality of neuroanatomical asymmetries in the zebrafish forebrain. Neuron.

[8] Gamse J.T., Kuan Y.-S., Macurak M., Brösamle C., Thisse B., Thisse C., Halpern M.E. (2005). Directional asymmetry of the zebrafish epithalamus guides dorsoventral innervation of the midbrain target. Development.

[9] Hendricks M., Jesuthasan S. (2007). Asymmetric innervation of the habenula in zebrafish. J. Comp. Neurol..

[10] Miyasaka N., Morimoto K., Tsubokawa T., Higashijima S.-I., Okamoto H., Yoshihara Y. (2009). From the olfactory bulb to higher brain centers: genetic visualization of secondary olfactory pathways in zebrafish. J. Neurosci..

[11] Concha M.L., Russell C., Regan J.C., Tawk M., Sidi S., Gilmour D.T., Kapsimali M., Sumoy L., Goldstone K., Amaya E. (2003). Local tissue interactions across the dorsal midline of the forebrain establish CNS laterality. Neuron.

[12] Ahrens M.B., Orger M.B., Robson D.N., Li J.M., Keller P.J. (2013). Whole-brain functional imaging at cellular resolution using light-sheet microscopy. Nat. Methods.

[13] Kishimoto N., Asakawa K., Madelaine R., Blader P., Kawakami K., Sawamoto K. (2013). Interhemispheric asymmetry of olfactory input-dependent neuronal specification in the adult brain. Nat. Neurosci..

[14] Jetti S., Vendrell-Llopis N., Yaksi E. (2014). Spontaneous activity governs olfactory representations in spatially organized habenular microcircuits. Curr. Biol..

[15] Bianco I.H., Wilson S.W. (2009). The habenular nuclei: a conserved asymmetric relay station in the vertebrate brain. Philos. Trans. R. Soc. Lond. B Biol. Sci..

[16] Agetsuma M., Aizawa H., Aoki T., Nakayama R., Takahoko M., Goto M., Sassa T., Amo R., Shiraki T., Kawakami K. (2010). The habenula is crucial for experience-dependent modification of fear responses in zebrafish. Nat. Neurosci..

[17] Okamoto H., Agetsuma M., Aizawa H. (2012). Genetic dissection of the zebrafish habenula, a possible switching board for selection of behavioral strategy to cope with fear and anxiety. Dev. Neurobiol..

[18] Kennedy D.N., O’Craven K.M., Ticho B.S., Goldstein A.M., Makris N., Henson J.W. (1999). Structural and functional brain asymmetries in human situs inversus totalis. Neurology.

[19] Ihara A., Hirata M., Fujimaki N., Goto T., Umekawa Y., Fujita N., Terazono Y., Matani A., Wei Q., Yoshimine T. (2010). Neuroimaging study on brain asymmetries in situs inversus totalis. J. Neurol. Sci..

[20] Roussigné M., Bianco I.H., Wilson S.W., Blader P. (2009). Nodal signalling imposes left-right asymmetry upon neurogenesis in the habenular nuclei. Development.

[21] deCarvalho T.N., Akitake C.M., Thisse C., Thisse B., Halpern M.E. (2013). Aversive cues fail to activate fos expression in the asymmetric olfactory-habenula pathway of zebrafish. Front. Neural Circuits.

[22] Carl M., Bianco I.H., Bajoghli B., Aghaallaei N., Czerny T., Wilson S.W. (2007). Wnt/Axin1/beta-catenin signaling regulates asymmetric nodal activation, elaboration, and concordance of CNS asymmetries. Neuron.

[23] Chen B., Dodge M.E., Tang W., Lu J., Ma Z., Fan C.-W., Wei S., Hao W., Kilgore J., Williams N.S. (2009). Small molecule-mediated disruption of Wnt-dependent signaling in tissue regeneration and cancer. Nat. Chem. Biol..

[24] Frasnelli E. (2013). Brain and behavioral lateralization in invertebrates. Front. Psychol..

[25] Miklósi A., Andrew R.J., Savage H. (1997). Behavioural lateralisation of the tetrapod type in the zebrafish (Brachydanio rerio). Physiol. Behav..

[26] Stigloher C., Ninkovic J., Laplante M., Geling A., Tannhäuser B., Topp S., Kikuta H., Becker T.S., Houart C., Bally-Cuif L. (2006). Segregation of telencephalic and eye-field identities inside the zebrafish forebrain territory is controlled by Rx3. Development.

[27] de Borsetti N.H., Dean B.J., Bain E.J., Clanton J.A., Taylor R.W., Gamse J.T. (2011). Light and melatonin schedule neuronal differentiation in the habenular nuclei. Dev. Biol..

[28] Ribolsi M., Koch G., Magni V., Di Lorenzo G., Rubino I.A., Siracusano A., Centonze D. (2009). Abnormal brain lateralization and connectivity in schizophrenia. Rev. Neurosci..

[29] Riederer P., Sian-Hülsmann J. (2012). The significance of neuronal lateralisation in Parkinson’s disease. J. Neural Transm..

[30] Lister J.A., Robertson C.P., Lepage T., Johnson S.L., Raible D.W. (1999). nacre encodes a zebrafish microphthalmia-related protein that regulates neural-crest-derived pigment cell fate. Development.

[31] Gilmour D.T., Maischein H.M., Nusslein-Volhard C. (2002). Migration and function of a glial subtype in the vertebrate peripheral nervous system. Neuron.

[32] Westerfield M. (2000). The Zebrafish Book: A Guide for the Laboratory Use of Zebrafish (Danio rerio).

[33] Zhang L., Leung Y.F. (2010). Microdissection of zebrafish embryonic eye tissues. J. Vis. Exp..

[34] Ohki K., Chung S., Ch’ng Y.H., Kara P., Reid R.C. (2005). Functional imaging with cellular resolution reveals precise micro-architecture in visual cortex. Nature.

