# Supplementary material for: Left-Right Asymmetry Is Required for the Habenulae to Respond to Both Visual and Olfactory Stimuli
Source: Curr Biol. 2014 Feb 17;24(4):440–5. doi: 10.1016/j.cub.2014.01.016 (PMC3969106; doi:10.1016/j.cub.2014.01.016)
Supplement: Document S1. Figures S1–S3 [file mmc1.pdf]

Supplemental Information

Figure S1, related to Figure 1

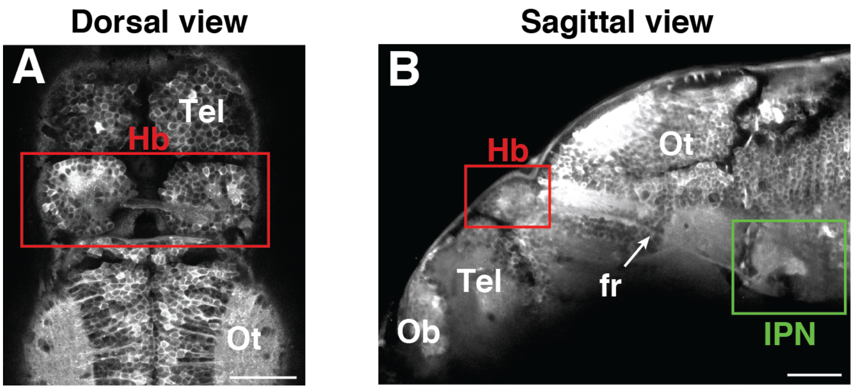

Figure S2, related to Figure 3

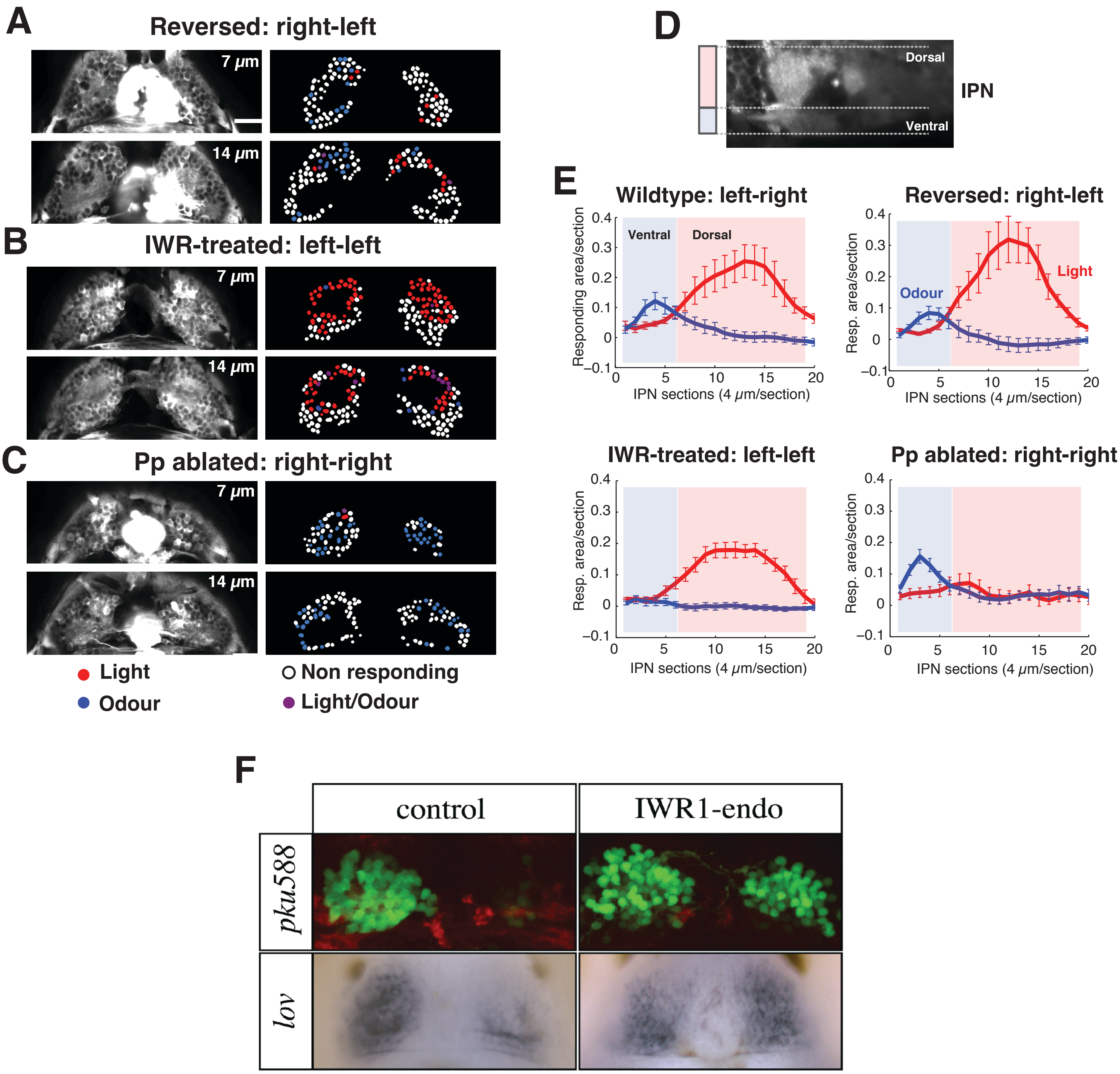

Figure S3, related to figure 4

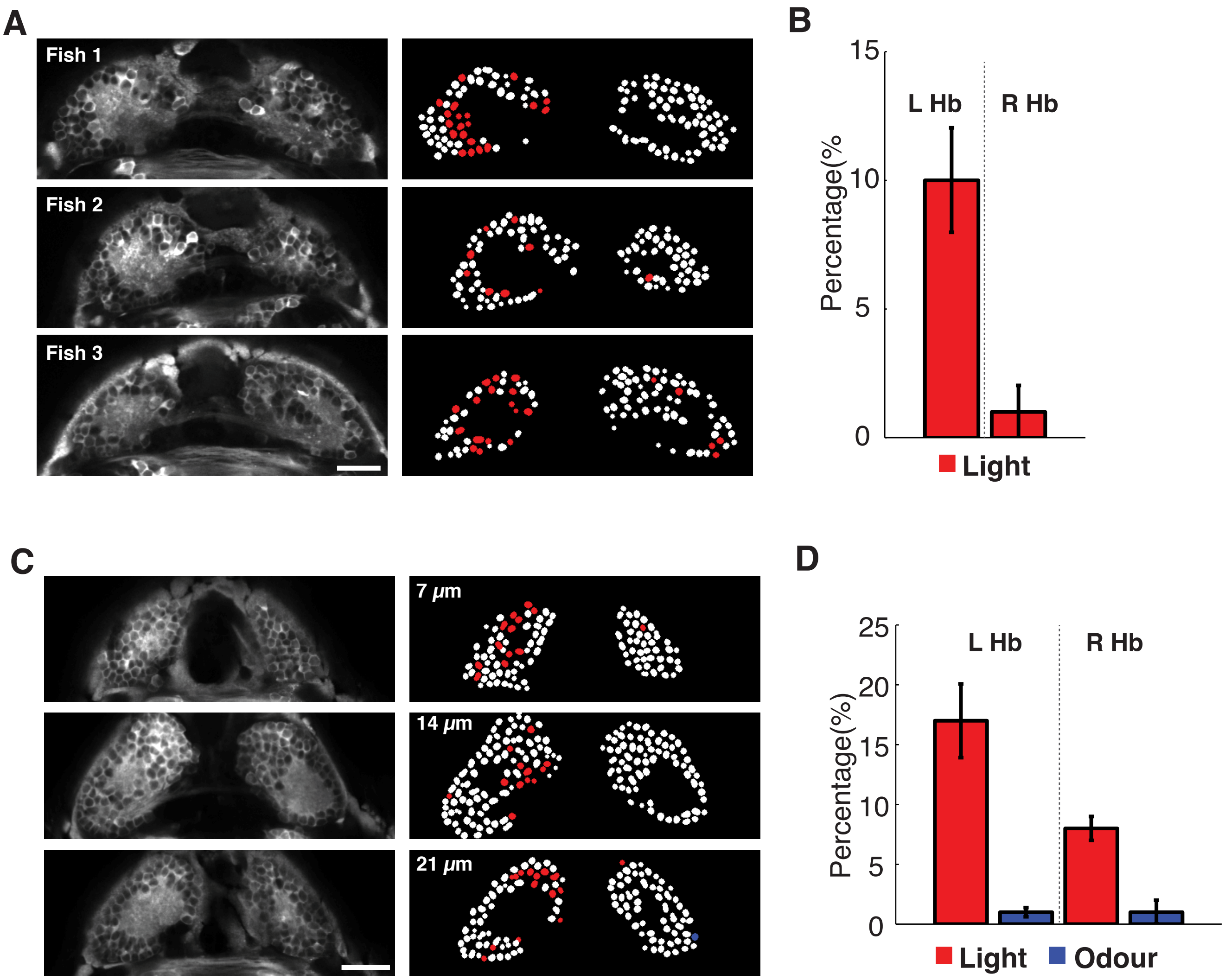

Figure S1, related to Figure1. Transgenic line used for imaging.
(A-B) Dorsal (A) and sagittal (B) views of a 4dpf Tg(*elavl3*:GCaMP5G) fish showing the Hb nuclei (red box) and the IPN (green box). Scale bar 50 μm. Abbreviations: Tel, telencephalon; Ob, olfactory bulb; Ot, optic tectum; Hb, habenulae; fr, fasciculus retroflexus; IPN, interpeduncular nucleus.

Figure S2, related to Figure 3. Functional responses to visual and odour stimulations in fish with reversed, double-left and double-right epithalami in the Hb and IPN.
(A-F) Raw images of two z-planes (at 7 and 14 μm depth) of the dorsal Hb of 4 dpf Tg(*elavl3*:GCaMP5G) reversed (A), double-left (B), and double-right (C) brained fish (left panels). In the right panels, dHb neuron cell bodies, indicated as regions of interests (ROIs), are colour-coded in red, blue, violet, or white dependent upon their response to light, odour, both, or neither respectively. In the case of parapineal reversal Tg(*elavl3*:GCaMP5G) fish were crossed with the Tg(*foxD3*:GFP) fish to visualize the parapineal on the right side (A). For the Parapineal (Pp) ablation, Tg(*elavl3*:GCaMP5G) fish were crossed with Tg(*foxD3*:GFP) and Tg(*flh*:GFP) fish (C). Scale bar 20 μm. (D) Sagittal view of the IPN highlighting the dorsal (light red) and ventral (light blue) IPN. (E) Depth profile of averaged light (red line) and odour (blue) IPN responses from dorsal to ventral in wildtype, reversed, double-left, and double-right conditions. Red and blue lines are averages of three trials for each stimulus modality. (n=7 wildtype, n=6 reversed, n=8 double-left, and n=6 double-right). (F) Dorsal views of the epithalami of control (left) and IWR1 treated (right) embryos showing expression of markers normally lateralized to the majority of left habenular neurons. Top shows the *pku588* transgene (Lu Wen, Bo Zhang, Shou Lin, MC and SW, unpublished data) and B,B' shows *kcdt12.1* (Gamse et al., 2003) expression. IWR1-mediated suppression of Wnt-signalling leads to many right-sided habenular neurons expressing markers typical of the neurons on the left. This consequently leads to an overtly double-left phenotype.

Figure S3, related to Figure 4. Dark-reared fish and fish with olfactory inputs removed show no change in lateralization of functional sensory responses.
(A-D) Examples (A) of a two-photon images of a single planes of the dHb (at 14 μm depth), where the majority of light responses are localised, of three Tg(*elavl3*:GCaMP5G) 4 dpf dark- reared fish (left). Corresponding colour-coded calcium signals in response to a light stimuli (right). Light responding neurons are in red. Scale bar 20μm. (B) Bar chart showing the percentages of light responding neurons (normalized to the total number of dHb neurons) in the left and right dHb nuclei for two sampled planes (at depths of 7 and 14μm) in multiple dark-reared fish (number of fish=4). (C) Examples of two-photon images of three single planes of the dHb (7, 14 and 21 μm depth) of a Tg(*elavl3*:GCaMP5G) 4 dpf fish with nose pit removal at 1 dpf, before mitral cell projections reach the Hb, (left). Corresponding colour-coded calcium signals in response to non-lateralized presentation of light and

odour (right). Light responding neurons are in red, while odour responses in blue. Scale bar 20µm. (D) Bar charts showing the percentages of light (red) versus odour (blue) responding neurons (normalized to the total number of dHb neurons) in the left and right dHb nuclei for two sampled planes (at 7 and 14µm, number of fish=6). Functional asymmetry of light responses is still present after olfactory pit removal. Error bars are SEM.
